# Case report: Clinical highlights and radiological classification of IgG4-related spinal pachymeningitis: A rare case series and updated review of the literature

**DOI:** 10.3389/fonc.2022.1035056

**Published:** 2023-01-10

**Authors:** Fan Yang, Zhengang Liu, Yibo Zhang, Pengfu Li, Yuhang Zhu, Qingsan Zhu, Boyin Zhang

**Affiliations:** Department of Orthopaedics, China-Japan Union Hospital of Jilin University, Changchun, China

**Keywords:** immunoglobulin G4 (IgG4), immunoglobulin G4-related disease (IgG4-RD), immunoglobulin G4-related hypertrophic pachymeningitis (IgG4-RHP), immunoglobulin G4-related spinal pachymeningitis (IgG4-RSP), American Spinal Injury Association (ASIA), spinal compression, magnetic resonance imaging (MRI)

## Abstract

**Purpose:**

Hypertrophic pachymeningitis associated with immunoglobulin G4-related disease (IgG4-RD) has been rarely reported, and there is little information and no clear consensus on the management of IgG4-related spinal pachymeningitis (IgG4-RSP). The present study described its possible clinical features, including the symptoms, imaging, treatment and prognosis of patients with IgG4-RSP.

**Methods:**

We report three patients who presented with progressive neurological dysfunction due to spinal cord compression. Relevant articles were searched from the PubMed, Web of Science, and Embase databases, and the resulting literature was reviewed.

**Results:**

The literature review provided a summary of 45 available cases, which included three cases from our center. Progressive worsening of neurological impairment was observed in 22 patients (48.9%). The lesions involved the thoracic spine (*n*=28, 62.2%), cervical spine (*n*=26, 57.8%), lumbar spine (*n*=9, 20.0%), and sacral spine (*n*=1, 2.2%). Furthermore, the lesions were located in the dura mater (*n*=18, 40.0%), epidural space (*n*=17, 37.8%), intradural-extramedullary space (*n*=9, 20.0%), and intramedullary space (*n*=1, 2.2%). On magnetic resonance imaging (MRI), the lesions generally appeared as striated, fusiform, or less often lobulated oval changes, with homogeneous (*n*=17,44.7%) and dorsal (*n*=15,39.5%) patterns being the most common. Thirty-five patients had homogeneous T1 gadolinium enhancement. Early surgical decompression, corticosteroid treatment, and steroid-sparing agents offered significant therapeutic advantages. A good therapeutic response to disease recurrence was observed with the medication.

**Conclusion:**

The number of reported cases of IgG4-RSP remains limited, and patients often have progressive worsening of their neurological symptoms. The features of masses identified on the MRI should be considered. The prognosis was better with decompression surgery combined with immunosuppressive therapy. Long-term corticosteroid treatment and steroid-sparing agent maintenance therapy should be ensured. A systemic examination is recommended to identify the presence of other pathologies.

## Introduction

IgG4 is a subtype of the immunoglobulin IgG that accounts for approximately 1-4%, and IgG4-related disease (IgG4-RD) is closely associated with IgG4 lymphocytes as an antigen-driven disease (1–4). The discovery of IgG4-RD has been a gradual process. Hamano et al. (2001) were the first to report that autoimmune pancreatitis is associated with elevated serum IgG4 (1). Furthermore, Kamisawa et al. (2) identified various IgG4-positive plasma cells in pancreatic biopsies with characteristic changes, such as interstitial fibrosis and obliterative phlebitis, which confirmed the presence of IgG4-RD. Subsequent studies have revealed that IgG4-RD is a systemic immune-mediated inflammatory disease of fibrosis (3, 4). The most common sites of involvement include the pancreas, bile duct, salivary glands, lacrimal glands, posterior peritoneum wall, and lymphatic capillaries (5), and the peripheral and central nervous system may also be involved (6). Some researchers have reported that IgG4-related peripheral neuropathy primarily involves the orbital or paravertebral region, and that this is often incidentally detected on images of other involved organs (7). IgG4-related hypertrophic pachymeningitis (IgG4-RHP) is a rare complication that involves the dura mater, which results in cranial neuropathy, encephalitis, hypophysitis and inflammatory pseudotumor (8). Chan et al. (9) reported the first case of IgG4-related spinal pachymeningitis (IgG4-RSP) with spinal cord compression in a 37-year-old male. IgG4-RSP can cause spinal compression that mimics metastatic disease or hemorrhage, and this condition is frequently misdiagnosed. The infiltration of various plasma cells in histopathological sections is a characteristic of IgG4-RSP (10). The present study presents three cases of IgG4-RSP, and discusses the clinical symptoms, radiological findings, treatment and prognosis. A review of the literature on this topic was also presented. In addition, The imaging-based typing of IgG4-RSP was attempted using radiological findings and lesion sites, in order to further improve the diagnosis and treatment of this disease.

## Materials and methods

### Case reports

#### Case 1

A 68-year-old male with no past medical history was admitted with a three-day history of incontinence and paraparesis. At four weeks prior to presentation, the symptoms began with lower extremity numbness, fatigue, and difficulty in walking. The patient’s physical examination on admission revealed loss of pain, light tactile, and temperature sensations below T10. The strength of each muscle group of the bilateral lower extremities was graded as 0. Furthermore, deep tendon reflexes, cremaster and anal reflexes were absent, and the toes were downgoing (American Spinal Injury Association, ASIA Grade A). Moreover, the plantar stimulation bilaterally elicited a flexion response (negative Babinski sign).

The laboratory examination revealed normal whole blood cell count, erythrocyte sedimentation rate (ESR), C-reactive protein (CRP) level, electrolyte concentrations, and hepatic and renal function. Serum IgG and IgG4 were not measured.

**Figure 1:**
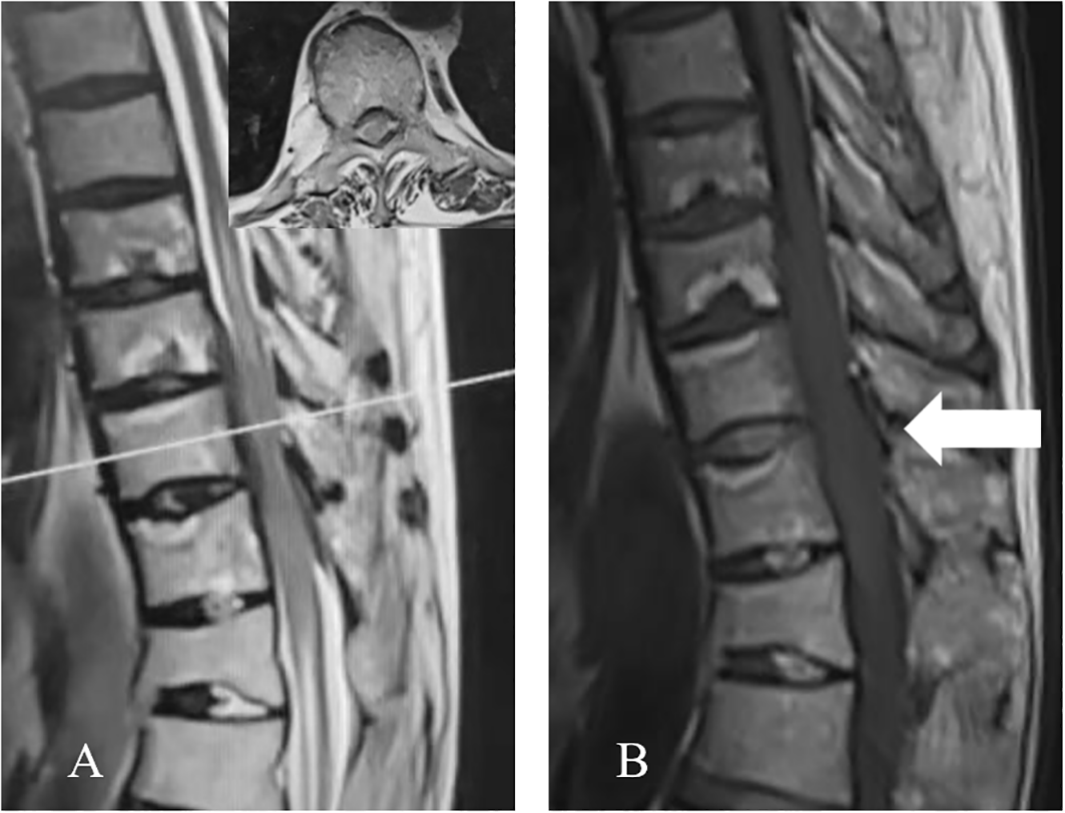
Preoperative thoracic MRI. **(A)** The sagittal T2WI revealed the homogeneous anterior-posterior spinal cord compression at the T9-T11 level, with a hypointense signal. One to two segments of the head and tail of the lesion presented cavitary changes in the spinal cord. The axial T2WI (corresponding to the white line in **A**) revealed the ventral and dorsal thickening of the dura, with a hypointense signal. **(B)** The sagittal T1WI presents the hypointense signal (arrow).

The thoracic magnetic resonance imaging (MRI) revealed abnormal band-like signals at the anterior-posterior margin of the dura at the T9-11 level. Hypointense signals were revealed on the T1-weighted imaging (T1WI) and T2-weighted imaging (T2WI). One to two segments of the head and tail of the lesion revealed cavitary changes in the spinal cord. The spinal cord was primarily compressed at the ventral border, which resulted in a decreased anterior-posterior diameter. The bony structures and paravertebral soft tissues were not involved (**Figure 1**).

The patient was preoperatively diagnosed with space-occupying lesions, and tumors or hematomas were highly suspected. On the day of admission, intravenous dexamethasone (20 mg) was administered, and an indwelling catheter was installed. Considering the severe neurological symptoms, the enhanced MRI was waived, and emergency surgery was performed.

**Figure 2:**
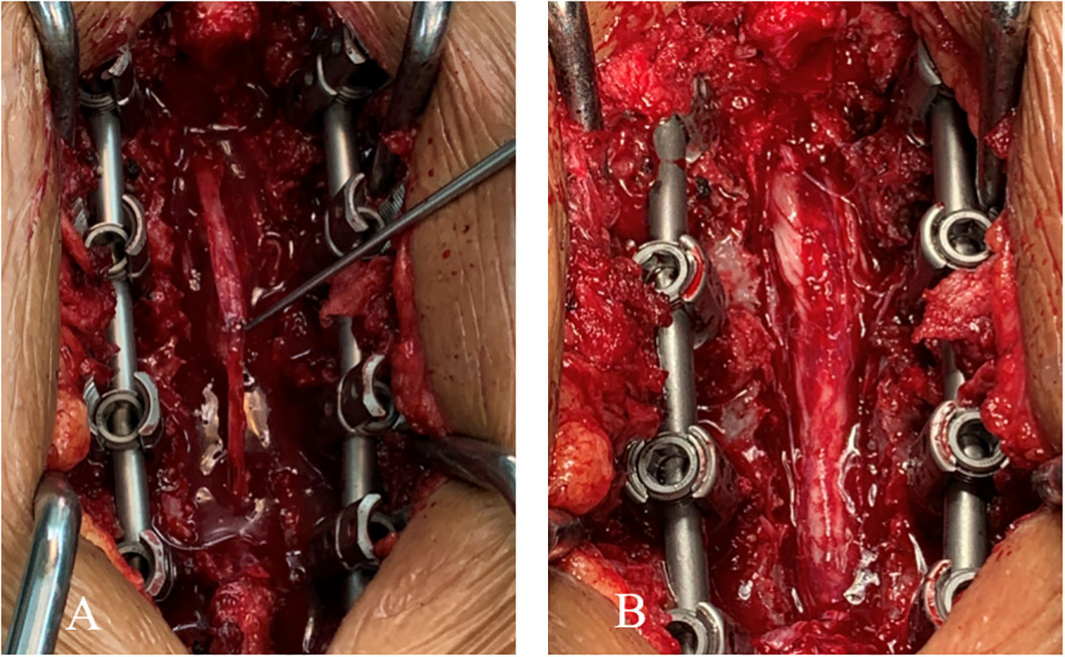
**(A)** The thickened dorsal dura presenting a band-like shape and rubbery texture (approximately 5.0 cm in diameter). **(B)** View of the surgical site after the complete resection of the thickened dorsal dura.

Laminectomy was initially performed at the T9-T12 vertebral levels. The dorsal dura presented with band-like thickening, a rubbery texture, and a lesion of approximately 5.0 cm. Furthermore, there were unclear boundaries within the normal dura. The thickened dorsal dura was completely resected. However, the structures of the arachnoid mater and pia mater were difficult to define. The purple spinal cord became thinner, and did not pulsate. The dura mater was closed after the absence of spinal canal space-occupying residues was confirmed. The resected thickened dura was processed for biopsy (**Figure 2**).

**Figure 3:**
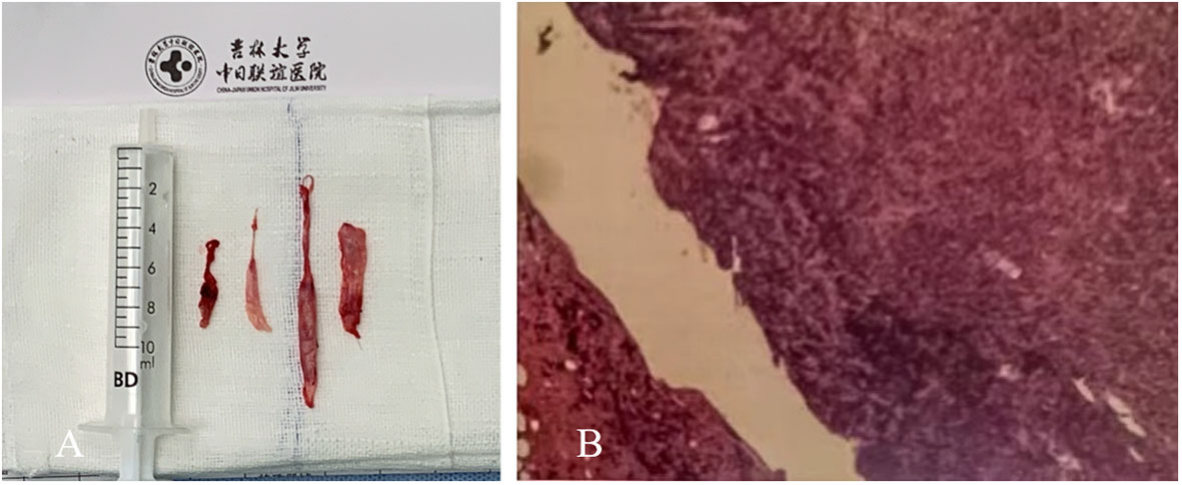
**(A)** A rubbery lesion resembling fish meat. **(B)** On histopathological examination, the dural mass and surrounding soft tissues presented with chronic and acute inflammation, inflammatory granulomatous micro-abscesses, and a few granulomas. Fibrous tissue hyperplasia, calcification, and a large number of plasma cells were present. The immunohistochemistry revealed the large-scale infiltration of IgG4-positive plasma cells.

The histopathological examination revealed fibrosing inflammatory changes in the dura due to the infiltration of large numbers of plasma cells and fibrous tissue hyperplasia. The immunohistochemistry suggested an IgG4-related autoimmune disease, which led to the final diagnosis of IgG4-RSP (**Figure 3**).

Postoperatively, dexamethasone (20 mg, *i.v.*) was administered once, daily, for two consecutive weeks. During the drug course, the muscle strength of the bilateral lower extremities remained at Grade 0, and there was no improvement in the patient’s sensory deficits or incontinence. The patient refused to undergo MRI re-examination and IgG4 serology tests due to economic factors, which resulted in the lack of postoperative radiological and serological results. The patient was discharged on day 14, postoperatively, with improved urination and sphincter function (ASIA grade B). The patient underwent rehabilitation therapy and symptomatic treatment in a local hospital after discharge, and concomitant oral treatment with 60 mg/d of prednisone for one month. The dose was reduced to 40 mg/d after one month, and maintenance treatment was continued for two months. The drug dose was slowly further reduced over a period of three months. After six months, the lower extremity muscle strength was grade III-IV. The patient had normal sensory functions and improved defecation, and could walk.

#### Case 2

**Figure 4:**
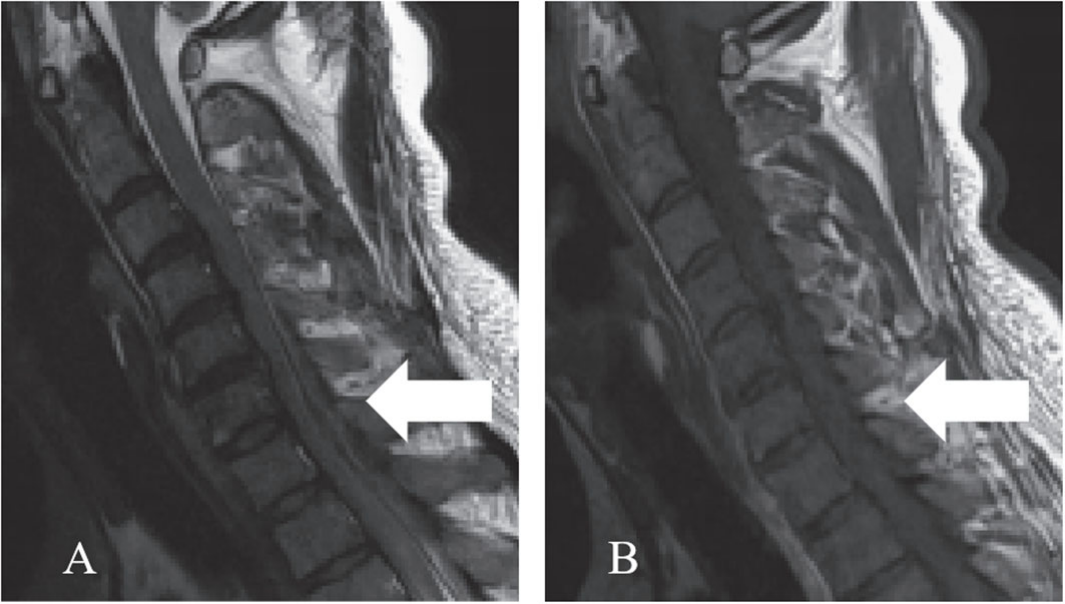
Preoperative cervical MRI. **(A)** The sagittal T2WI revealed a slightly hyperintense signal (arrow). **(B)** The sagittal T1WI revealed a hypointense signal (arrow).

A 43-year-old male visited our hospital with a 15-day history of neck pain, and bilateral lower-extremity weakness and incontinence for four days as the chief complaints. The physical examination upon admission revealed loss of superficial sensation below T12, grade III triceps brachii strength of the bilateral upper extremities (grade IV for the remaining muscles), grade II muscle strength of the main muscle groups of the bilateral lower extremities, and positive bilateral pathological signs (ASIA grade C). The serological tests revealed a serum IgG level of 9.76 g/L, which was within the normal range (8.00-16.00 g/L). The MRI manifestations included a strip-shaped mass in the dorsal dura at the C4-T2 level, a slightly hyperintense signal on T2WI, and a hypointense signal on T1WI (**Figure 4**).

An indwelling urinary catheter was placed for the patient after admission. Before surgery, the patient presented with progressive symptoms, including loss of superficial sensation below T4, grade 0 muscle strength in each muscle group of the bilateral lower extremities, and loss of sensation (ASIA grade A). Epidural hematoma was considered, methylprednisolone (1,000 mg, *i.v.*) was administered, and emergency surgical decompression was applied. A rubbery, strip-shaped mass that resembled fish meat was tightly adhered to the dorsal dura at the C2-T3 level, and the epidural space was completely compressed without any hematomas. The mass was completely resected and sent for pathological examination. The results revealed diffuse lymphocyte infiltration with a large number of mature plasma cells. Active fibrohistiocytic proliferation was also visible in the mesenchyme. The immunohistochemical staining and quantification of IgG4 in high-power fields (HPFs) revealed that greater than 40% of the cells were IgG-positive cells (IgG4/IgG >40). Hence, a diagnosis of IgG4-RSP was made. Postoperatively, the patient was intravenously treated with 240 mg/d of methylprednisolone, and the patient experienced improvements in sensory function of the lower extremities beginning on day five. On day 14, the patient was discharged, but the muscle strength of each muscle group in the bilateral lower extremities remained at 0 (ASIA grade B). After discharge, the patient was instructed to orally take 40 mg/d of prednisone for two months. The dose of prednisolone was slowly reduced over the next four months. During the six-month follow-up, the muscle strength of the main muscle groups in the bilateral lower extremities recovered to level IV, and the sensory function returned to normal.

#### Case 3

A 39-year-old male presented for treatment due to a 15-day history of back pain, a two-day history of lower extremity weakness, difficulty in walking, and difficulty in urinating. The patient was admitted to our hospital. The patient’s physical examination on admission revealed tenderness of segment T2, loss of sensation below T12, grade III muscle strength of the main muscle groups of the bilateral lower extremities, and positive bilateral pathological signs (ASIA grade D). The cerebrospinal fluid (CSF) collected *via* lumbar puncture revealed an IgG level of 718.0 mg/L. The cervical MRI revealed abnormal strip-shaped regions of signals at the anterior-posterior margin of the dura at the C5-T4 level, a hypointense signal on T1WI, a hyperintense signal on T2WI, and homogeneous enhancement after gadolinium enhancement on T1WI. Methylprednisolone (1,000 mg/d) was intravenously administered as a pharmacological treatment after admission. However, the patient’s symptoms worsened the following day, and presented with loss of sensation below the horizontal line of the bilateral costal arch, and a decrease to grade II (ASIA grade C) in muscle strength of the bilateral lower extremities. An extramedullary hematoma or nodule was highly suspected. Therefore, methylprednisolone was discontinued on day three, and anti-tuberculous therapy was performed. After five days, the muscle strength of the bilateral lower extremities was graded as 0 (ASIA grade A). The MRI revealed abnormal strip signals in the anterior-posterior margin of the dura at the T1-T6 level, a hypointense signal on sagittal T1WI, a hyperintense signal on T2WI, and homogeneous gadolinium enhancement. Emergent surgical decompression was immediately performed. Rubbery, strip-shaped masses with a hard texture were widely distributed in the dura mater. The subdural and epidural lesions were resected and sent for pathological examination. The results revealed diffuse plasma cell infiltration in fibro-adipose tissues. The HPFs of the immunohistochemically stained slides revealed that greater than 40% of cells were IgG positive (IgG4+/IgG+ in >10 HPFs). The serological tests revealed a serum IgG4 level of 1.050 g/L. The patient was eventually diagnosed with IgG4-RSP. Methylprednisolone (500 mg/d) was postoperatively administered, and the muscle strength of the right lower extremity recovered to grade 1. The dose of methylprednisolone was reduced to 240 mg/d for six consecutive days, and reduced to 120 mg/d for three consecutive days. The muscle strength of the right lower extremity recovered to grade 2, and the left lower extremity returned to grade 1. However, there was no improvement in the patient’s sensory function (ASIA grade C). After discharge, the patient was prescribed with oral prednisolone of 40 mg/d for two months, followed by a gradual dose reduction over a three-month period. During the long-term follow-up period, the motor and sensory functions of the upper extremities progressively recovered, and there was no evidence of recurrence on MRI.

## Literature review

**Figure 5:**
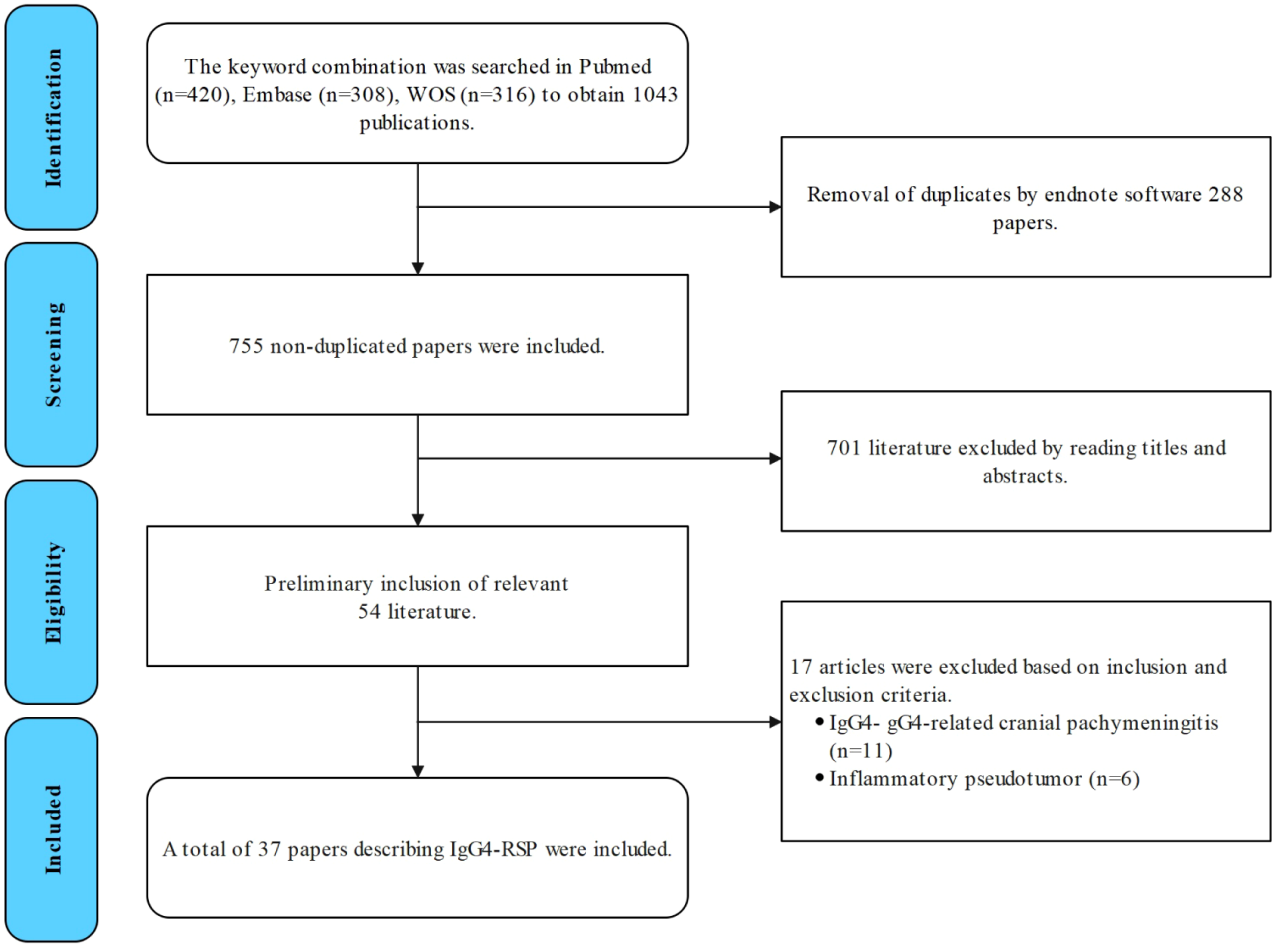
Flowchart for the literature search for IgG4-RSP.

The studies were retrieved from the PubMed, Web of Science, and Embase databases using the following keywords as search terms: (IgG4-related disease) and (IgG4-related sclerosing pachymeningitis OR IgG4-related spinal pachymeningitis OR IgG4-related hypertrophic pachymeningitis) and (pachymeningitis) and (spine OR spinal). Case reports, case series, and single-center and multicenter retrospective studies that investigated IgG4-related spinal lesions were included. Studies that only involved IgG4-related cranial or inflammatory pseudotumors were excluded (**Figure 5**).

Two evaluators selected the studies, and any disagreements were resolved by negotiation or consulting with the corresponding author. The following information was extracted from each of the selected articles: author’s name, patient’s age, patient’s gender, clinical presentations, location of mass involvement, MRI features, immunohistochemical findings, treatment outcome, and prognosis.

## Statistical analysis

The data was analyzed using the SPSS 22.0 statistical software. Count data were expressed as *n* [%]. Comparisons between groups were performed by chi-square test, totals of less than 40 were compared using Fisher’s exact test, and multiple comparisons were performed using Bonferroni correction, α=0.05. *P*<0.05 was considered statistically significant.

## Results

The selection process is illustrated in **Figure 5**. A total of 37 studies were included for the analysis in the present study, with a total of 45 patients, including the three patients reported in the present study (the details are provided in **Table 1**) (8–44). The present study examined the clinical symptoms, radiological manifestations, treatment and recurrence of IgG4-RSP.

**Table 1:** Clinical data of the 45 cases of IgG4-RSP.

Study	Age and gender	Involved area	Imaging Features	Treatment	Outcome (After the first treatment)	Recurrence (During the follow-up period)
Chan et al. (9)	37 Male	T5-T10. (Epidural) (Bilateral submandibular gland involvement)	Dorsal type T2: hyperintense. T1: homogeneous gadolinium enhancement	Decompression surgery	N/A	N/A
Choi et al. (11)	46 Female	T9-T11. (Epidural)	Homogeneous type. T2: hypointense. T1: homogeneous gadolinium enhancement (T9-T11, epidural surround)	Decompression surgery, corticosteroids and antituberculous medication.	Improved	At two months after surgery.
Lindstrom et al. (12)	55 Male	C3-7. (Dural)	N/A	N/A	Improved	No
	63 Male	C2-3. (Dural)	N/A	N/A	N/A	N/A
Della-Torre et al. (13)	65 Male	Posterior cranial fossa to C4 (Dural) (hypertrophic pachymeningitis of the posterior cranial fossa and abdominal periaortitis)	Dorsal type T1: homogeneous gadolinium enhancement	Corticosteroids, methotrexate and cyclophosphamide	Improved	No
Tajima et al. (14)	64 Male	T3-T11 (Epidural) (Cranial dura and renal involvement)	Dorsal type T1: homogeneous gadolinium enhancement	Corticosteroids	Improved	No
Wallace et al. (15)	32 Male	L5 (Dural)	N/A	Decompression surgery	Improved	N/A
Della-Torre et al. (16)	48 Female	Cranial dura to C1 (Dural) (Involvement of the posterior cranial fossa and greater occipital foramen)	Dorsal type T1: homogeneous gadolinium enhancement	Corticosteroids and cyclophosphamide	Improved	No
Kim et al. (17)	52 Female	C7-T5 (Intradural)	Ventral type T2: hypointense. T1: homogeneous gadolinium enhancement	Decompression surgery and corticosteroids	Improved	No
Sakai et al. (18)	32 Female	C1-7, L5-S1 (Dural) (Cranial dura, pituitary, and lungs involvement.)	Dorsal type. T1: homogeneous gadolinium enhancement.	Corticosteroids	Improved	No
Zwicker et al. (19)	58 Female	C7-T6 (Epidural and dural)	Homogeneous type T1: hypointense. T2: hypointense. T1: homogeneous gadolinium enhancement	Decompression surgery, corticosteroids and methotrexate	Improved	No
Chen et al. (20)	49 Male	T1-T3,L4-L5 (Epidural)	Homogeneous type. T1: homogeneous gadolinium enhancement	Decompression surgery, corticosteroids and antituberculous medication	Improved	A mass of subcutaneous tissue on the left side of the neck was found at eight months after surgery.
Ezzeldin et al. (21)	55 Male	T2−T3 (Epidural and paraspinal)	Ventral type T2: hypointense. T1: homogeneous gadolinium enhancement	Decompression surgery and corticosteroids	Improved	N/A
Lu et al. (8)	55 Male	C2-T9 (Dural)	Homogeneous type T1: homogeneous gadolinium enhancement	Decompression surgery, corticosteroids and cyclophosphamide	Improved	No
Ferreira et al. (22)	57 Female	T10-T12. (Epidural)	Homogeneous type T1: homogeneous gadolinium enhancement	Decompression surgery	Improved	At three weeks after the first surgery and two months after the second surgery.
Radotra et al. (23)	50 Male	L1-L2 (Intradural)	Homogeneous type T1: isointense. T2: hypointense T1: homogeneous gadolinium enhancement	Decompression surgery and corticosteroids.	Improved	No
	19 Male	L2-L3 (Intradural)	Homogeneous type T1: isointense. T2: hypointense. T1: homogeneous gadolinium enhancement	Decompression surgery and corticosteroids	Improved	No
Yangue et al. (24)	18 Male	C1-C7 (Intradural)	N/A	Decompression surgery	Improved	N/A
Zhao et al. (25)	49 Female	T1-T4 (Epidural)	Dorsal type T1: hyperintense T2: hyperintense T1: homogeneous gadolinium enhancement	Decompression surgery	N/A	N/A
Fernández-Codina et al. (26)	55 Male	Cranial dura to C3 (Dural) (Cranial dura, pleura and lungs involvement)	Homogeneous type T1: homogeneous gadolinium enhancement	Pleuro-pulmonary (biopsy), corticosteroids and rituximab	Improved	No
Williams et al. (27)	46 Female	C4-T1. (Epidural and paraspinal)	Homogeneous type T1: hypointense T1: homogeneous gadolinium enhancement	Corticosteroids and azathioprine	Improved	No
Maher et al. (28)	79 Female	C6-L2. (Dural)	Dorsal type. T1I: hypointense T2: hypointense T1: homogeneous gadolinium enhancement	Decompression surgery, corticosteroids and rituximab	Improved/Died from infection	No
Bridges et al. (29)	68 Male	T3-T5. (Intradural)	Ventral type T1: hypointense T2: hypointense T1: homogeneous gadolinium enhancement	Decompression surgery and corticosteroids	Improved	No
Rumalla et al. (30)	50 Male	T4-T6 (Epidural and paraspinal) (Lungs involvement)	Ventral type T1: homogeneous gadolinium enhancement	Decompression surgery and corticosteroids	Improved	No
Varrassi et al. (31)	62 Male	C4 -T1. (Intradural) (Cranial dura and orbital involvement)	Homogeneous type T1: hypointense T2: hyperintense T1: homogeneous gadolinium enhancement	Orbital biopsy and corticosteroids	Improved	No
Winkel et al. (32)	48 Female	L2−L3. (Epidural)	Homogeneous type T2: hypointense. T1: homogeneous gadolinium enhancement	Decompression surgery and corticosteroids	Improved	No
Cação et al. (33)	56 Female	Thoracic and lumbar spine (Dural)	Ventral type T2: hypointense T1: homogeneous gadolinium enhancement	Corticosteroids	Improved	Recurred within two months of the patient’s corticosteroid course
Levraut et al. (34)	55 Male	C3-T3 (Intradural)	Dorsal type T1: isointense T2: hypointense T1: homogeneous gadolinium enhancement	Decompression surgery and corticosteroids	Improved	No
Merza et al. (35)	60 Female	T1-T7 (Epidural)	Homogeneous type T1: homogeneous gadolinium enhancement	Decompression surgery and corticosteroids	Improved	No
Sireesha et al. (36)	40 Female	C5-T6 (Dural)	N/A	Corticosteroids	Improved	No
Slade et al. (37)	50 Male	C7-T5 (Epidural)	Dorsal type T2:hypointense	Corticosteroids	Improved	Recurrence after two months of the patient’s corticosteroid course
Melenotte et al. (38)	57 Male	Cervical spine (Epidural)	Dorsal type T1: homogeneous gadolinium enhancement	Decompression surgery and corticosteroids	Improved	No
	57 Male	T2-T3 (Dural)	N/A	Decompression surgery	Improved	No
	68 Male	T9-L2 (Epidural)	N/A	Decompression surgery, corticosteroids and rituximab	Improved	No
	31 Male	C3-C5 (Dural) (Cranial dura and orbital involvement)	Dorsal type T1: homogeneous gadolinium enhancement	Corticosteroids and rituximab	Improved	No
Vakrakou et al. (39)	17 Female	C2-C5 (Intramedullary) (Deep white matter and hypophysis involvement)	Homogeneous type T2: hyperintense. T1: homogeneous gadolinium enhancement	Corticosteroids and azathioprine	Improved	No
Li et al. (40)	58 Female	T2-T7 (Intradural)	Ventral type T1: hypointense T2: hypointense T1: homogeneous gadolinium enhancement	Decompression surgery.	Improved / Death from infection after recurrence.	Recurrence after the first postoperative year and at 3 months after the second.
Sbeih et al. (10)	24 Male	C7-T6 (Dural)	Dorsal type T1: hypointense T2: hypointense T1: homogeneous gadolinium enhancement	Decompression surgery and corticosteroids.	Improved	No
Elmaci et al. (41)	37 Female	C2-T2/T3 (Epidural)	Dorsal type T1: isointense T2: hypointense T1: homogeneous gadolinium enhancement	Decompression surgery.	Improved	Recurrence at two years after surgery.
Woo et al. (42)	62 Male	C4/C5 (Intradural)	Dorsal type T1: isointense T2: hyperintense T1: homogeneous gadolinium enhancement	Decompression surgery, corticosteroids, antituberculous medication and azathioprine.	Improved	No
Sharma et al. (43)	27 Male	Cranial dura to C6 (Dural) (Brain and orbits involvement)	Homogeneous type T1: isointense T2: iso- to hypointense. T1: homogeneous gadolinium enhancement	Decompression surgery, corticosteroids, azathioprine and rituximab	Improved	No
Sankowski et al. (44)	56 Male	Foramen magnum down to the C7 (Dural)	Homogeneous type T1: hypointense T2: hypointense T1: homogeneous gadolinium enhancement	Biopsy	N/A	N/A
The present case	68 Male	T9-T11 (Dural)	Homogeneous type T1: hypointense T2: hypointense	Decompression surgery and corticosteroids	Improved	No
	43 Male	C4-T2 (Epidural)	Dorsal type T1: hypointense T2: hyperintense	Decompression surgery and corticosteroids	Improved	No
	39 Male	C5-T4 (Epidural and Intradural)	Homogeneous type T1: hypointense T2: T2: hyperintense T1: homogeneous gadolinium enhancement	Decompression surgery, corticosteroids and antituberculous medication	Improved	No

## Clinical presentations

There were 29 males (64.4%) and 16 females (35.6%), with a male-to-female ratio of 1.8:1.0, and the age of most of the patients ranged between 40 and 70 years old (range: 17-79 years old, median age: 52). The thoracic spine (*n*=28, 62.2%) was the most frequently involved segment, followed by the cervical spine (*n*=26, 57.8%), lumbar spine (*n*=9, 20.0%) and sacral spine (*n*=1, 2.2%). Longitudinal extensive involvement across multiple spinal cord regions occurred in 11.1% of cases. The main symptoms were derived from the spinal cord compression in the involved segments, and these primarily presented as varying degrees of sensorimotor dysfunction of the extremities. Progressive deterioration of spinal cord compression symptoms was reported in 22 patients (48.9%). Headache and facial pain were reported in four patients (8.9%) who had a lesion between the brain and cervical spine. Eleven patients (24.4%) had a combined IgG4-related disorder with the involvement of other organs, such as the brain, which was the most commonly involved secondary organ.

## Radiological manifestations

**Figure 6:**
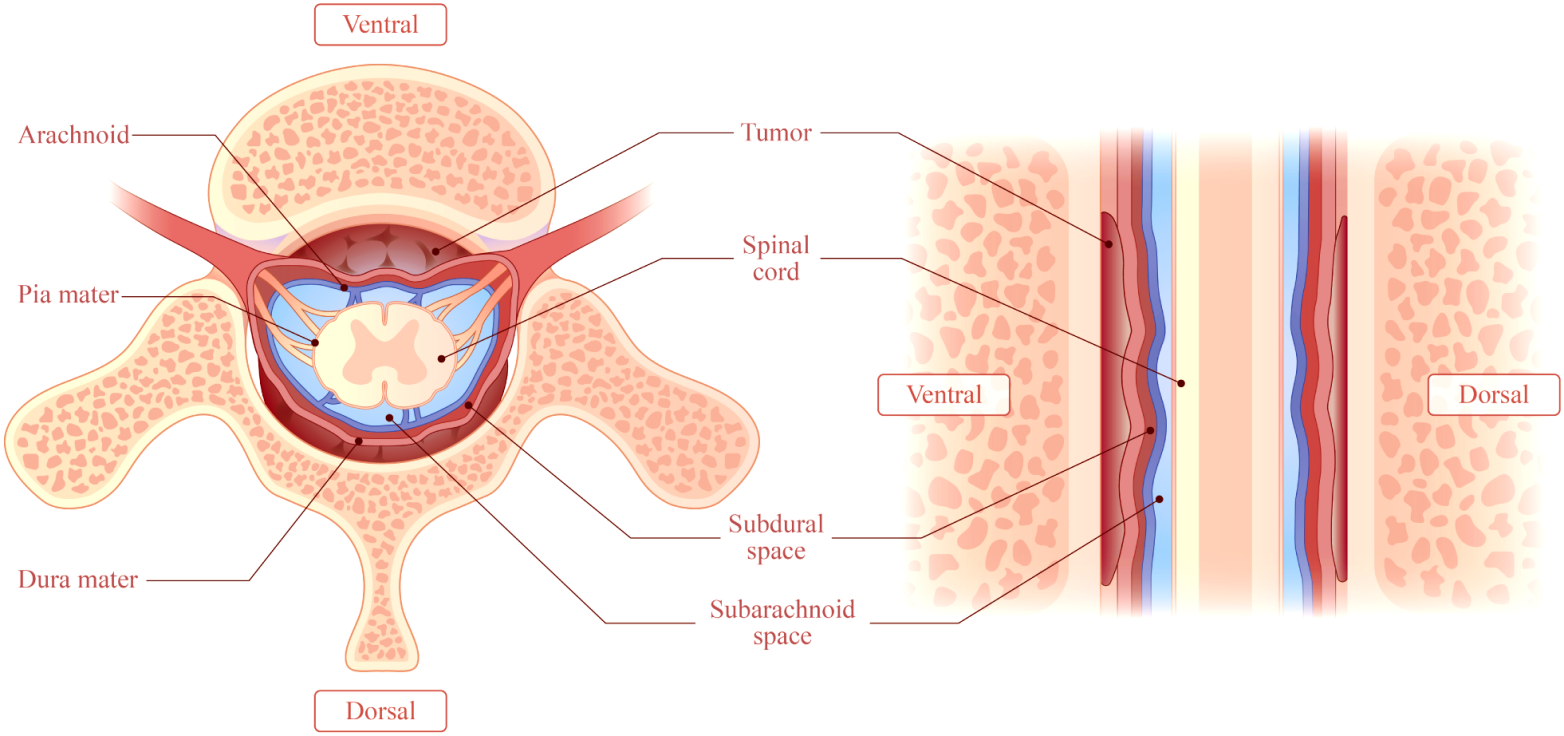
This illustrates a mass accumulating outside the dura. The mass may be classified as ventral, dorsal, or homogeneous (accumulation on the ventral and dorsal sides), depending on the location of the mass relative to the spinal cord in a right sagittal plane.

The lesion sites included the intradural-extramedullary (*n*=9, 20.0%), dural (*n*=18, 40.0%), epidural (*n*=17, 37.8%) and intramedullary (*n*=1, 2.2%). Five patients (11.1%) had multiple regions of involvement, including the epidural+paravertebral (*n*=3, 6.7%), dural+epidural (*n*=1, 2.2%), and epidural+intradural-extramedullary (*n*=1, 2.2%) regions. The dural and epidural spaces were the most commonly involved in IgG4-RSP. According to the reported sagittal MRI scans, the masses were defined as ventral, dorsal, or homogeneous (ventral and dorsal), based on the position relative to the spinal cord (**Figure 6**).

The lesions involved the dorsal cord in 15 patients (33.3%), the ventral cord in six patients (13.3%), and the homogeneous in 17 patients (37.8%), while this was not reported in seven patients.

In investigating the relationship between the location of the lesions and disease progression, it was identified that 82.3% (14) of 17 homogeneous patients were progressive. Meanwhile, 26.7% (4) of 15 dorsal patients and 66.7% (4) of six ventral patients were progressive. Furthermore, there was a significant difference in the progression rate between the three groups (*P*=0.004, *P*<0.01), and the results for the multiple comparison revealed that the progression rate was significantly higher in the homogeneous samples, when compared to the dorsal samples.

A total of 17 patients had T1WI data from the MRI: a hypointense signal on T1WI was observed in 10 patients, an isointense signal was observed in six patients, and a hyperintense signal was observed in one patient. Furthermore, the MRI T2WI data was obtained for the other 23 patients: a hyperintense signal on T2WI was observed in seven patients, a hypointense signal was observed in 16 patients, and an isointense-to-hypointense signal was observed in one patient. For all 35 patients, homogeneous enhancement of T1W1 with gadolinium was identified.

## Treatment and prognosis

The treatment regimen used for the initial treatment was examined (except for the two patients, in which no treatment plan was reported). Among these patients, 30 patients (66.7%) underwent decompressive laminectomy, 34 patients (75.6%) received immunosuppressive therapy, three patients (6.7%) underwent tissue biopsy at different sites, and four patients (8.9%) received empirical antituberculosis therapy.

The treatment regimen in the acute phase (*i.e.* initial disease detection) included surgical decompression and immunosuppressive therapy. Eight patients (19.0%) underwent surgical decompression only, six patients (14.3%) received corticosteroid therapy only, 16 patients (38.1%) underwent surgery combined with corticosteroid therapy, six patients (14.3%) received corticosteroids plus steroid-sparing agents, and six patients (14.3%) underwent surgery combined with corticosteroid plus steroid-sparing agent maintenance therapy. A long-term treatment (treatment cycle of >1 month) regimen was used to prevent recurrence, and focus was given on the long-term application of corticosteroids and steroid-sparing agents. Among these patients, 14 patients (53.8%) received long-term maintenance therapy with corticosteroids, and 12 patients (46.2%) received maintenance therapy with corticosteroids and steroid-sparing agents.

No treatment outcome was reported in four patients. For the remaining 41 patients, all patients presented with symptomatic relief and neurological recovery after initial treatment. Two of the patients (4.9%) died postoperatively due to infection after receiving initial treatments of surgical treatment alone, and medication combined with surgical treatment.

## Recurrence

Thirty-seven patients were followed up for longer than two months after the initial treatment. For these cases, a total of six relapses were reported (16.2%). The sub-analysis of various types of treatments revealed that three recurrences were observed in patients treated with decompression surgery alone (8.1%), two recurrences were observed in patients treated with corticosteroids only (5.4%), and one recurrence was observed in a patient treated with decompression surgery and corticosteroids (2.7%).

The specific duration of immunosuppressive therapy was not reported in two cases, while the remaining four cases did not experience prolonged immunosuppressive therapy at the time of initial treatment. Overall, no recurrences were observed in patients who received long-term treatment with immunosuppressives (*n*=26), and four recurrences were observed in patients who did not receive long-term therapies (*n*=9). The difference in recurrence rate between the two groups was statistically significant (*P*=0.002, *P*<0.01).

The treatment options after recurrence focused on surgery and immunosuppressive therapy. The specific treatments and prognoses for cases of recurrence are detailed in **Table 2**. Four patients had a single recurrence (10.8%). Among these patients, one patient had no reported specific treatment regimen, two patients received an increased dose of corticosteroid therapy, and one patient underwent decompression surgery combined with immunosuppressive therapy. Furthermore, two patients (5.4%) experienced secondary recurrence. These two patients only underwent decompressive surgical intervention without immunosuppressive therapy in the second round of treatment. For patients who developed recurrence, the results of the final follow-up revealed complete remission of neurological symptoms in two patients, partial remission in two patients, and death due to infection in one patient. No treatment outcome was reported for one patient.

**Table 2:** Treatment and prognosis of recurring patients.

Study	Initial treatment protocol	Treatment options after recurrence	Results after recurrence treatment	Presence of second recurrence and related treatment measures	Results after the second recurrence treatment
Choi et al. (11)	Decompression surgery combined with dexamethasone	Prednisone of 40 mg/d, lasting for one month, tapering to several months; empirical antituberculous medication	Complete remission	No	Full remission
Ferreira et al. (22)	Decompression surgery	Decompression surgery.	Partial remission	Yes Decompression surgery. Eight weeks of oral corticotherapy (1 mg/kg/d) and weekly epidural administration of methylprednisolone (80 mg/week).	Partial remission
Cação et al. (33)	Corticosteroids therapy (no details)	Increase in steroids/corticosteroids dose (no details)	Partial remission	No.	Full remission
Slade et al. (37)	Oral prednisone 40 mg/d for five days	Decompression surgery; 11 courses of prednisone or methylprednisolone Tapered; intravenous administration of rituximab of 375 mg/m^2^ weekly for four weeks.	Complete remission	No	Partial remission
Li et al. (40)	Decompression surgery	Decompression surgery	Partial remission	Yes Methylprednisolone and cyclophosphamide treatment for one month	Died of infection after nine months.
Elmaci et al. (44)	Decompression surgery	Not reported	Not reported	No	N/A.

Two patients with secondary recurrence were carefully reviewed. One patient was a 57-year-old female. The first recurrence occurred for this patient at three weeks after the initial decompression surgery. The symptoms were initially in remission after the second surgery. Notably, no immunosuppressive treatment was given during the follow-up period. A second recurrence developed at two months after the second surgery for this patient. The attending physician administered an eight-month course of oral prednisolone (1 mg/kg/d) and epidural injections of methylprednisolone acetate (80 mg/week) as maintenance therapy, in addition to decompression surgery. The follow-up results revealed a partial improvement of symptoms. A 58-year-old woman also underwent decompression surgery after first recurrence, and the symptoms improved. No immunosuppressive treatment was given for this patient after the surgery. This second patient developed recurrence after three months. The recurrence was managed with one month of methylprednisolone and cyclophosphamide, which provided symptom relief. Unfortunately, this patient eventually died from pulmonary infection.

## Discussion

### Symptoms

IgG4-RD involves multiple organ systems, and has a tendency to develop multiple neoplastic lesions. The clinical presentations are nonspecific, but these primarily consist of dysfunction of the involved organs or tissues (45, 46). Patients with IgG4-RD may also present with fever, fatigue, night sweats, and weight loss (47, 48). The most common symptoms in IgG4-RSP patients are fatigue and motor sensory dysfunction of the extremities, which occur in varying degrees. Among the 45 cases obtained for the present study, 22 patients experienced symptoms that gradually progressed over days to months, and these symptoms presented as severe myeloid symptoms (8, 10, 11, 17, 19, 20, 22, 23, 27, 29–31, 33–35, 39, 41, 43, 44). Therefore, the progressive exacerbation of neurological symptoms may be associated with a secondary spinal cord injury caused by chronic spinal cord ischemia, and the acute exacerbation of inflammatory cascade activation (49, 50). These progressively exacerbated neurological symptoms may serve as a hint during the clinical diagnosis.

### Immune molecular pathogenesis

The immune molecular pathogenesis of IgG4-RD has not been clarified. A previous research suggested that IgG4-RD is an antigen-driven disease that involves IgG4+ B cells and T cells (42). Della-Torre et al. (51) suggested an experimental pathogenic model of IgG4-RD after a literature review, and reported that B and T cells exert synergistic functions in chronic self-perpetuating immune reactions against specific antigens, during which the production of inflammatory factors IL-4 and IL-10 may drive antigen-specific B cells to undergo isotype switching, and secrete IgE and IgG4. Other reports have noted that macrophages and basophils are also potentially pathogenic for IgG4-RD (52, 53).

### Pathology, serology and CSF diagnosis

Hisanori et al. (2011) proposed the following presently well-accepted diagnostic criteria for IgG4-RD: (1) single or multiple organ thickening/nodular lesion, focal/diffuse enlargement or mass; (2) serum IgG4 of >135 mg/dL; (3) typical histopathological features. A definite diagnosis may be made when criterion (1), and criterion (2) or (3) are satisfied (54).

The typical histopathological features of IgG4-RD include intensive lymphocyte infiltration (IgG4/IgG+ cells >40% and/or IgG4+ plasma cells >10/HPF), storiform swirling fibrosis, and obliterative phlebitis (55). Obliterative phlebitis is rare in cases of IgG4-RSP (12, 23, 38). The present study revealed that 22 patients had IgG4/IgG+ of >40% (8–10, 12, 13, 22, 23, 25, 29, 34, 35, 38, 40, 42–44), and 33 patients had IgG4+ plasma cells of >10/HPF (8–12, 15, 19, 22–32, 34–38, 40, 42, 44). Although there is no international consensus criteria for the pathological diagnosis of IgG4-RSP, dura mater biopsy is presently the best-supported approach.

Inflammatory markers are non-specific for the diagnosis of IgG4-RD patients due to the differential presentations (56), and an increase in inflammatory indicators may be a result of infection and inflammation (57, 58). Therefore, the alterations in eosinophils, anti-nuclear antibodies and rheumatoid factors are also non-specific (59–61). Furthermore, most IgG4-RD patients have elevated serum IgG4 (>135 mg/dL), but there is no significant reference range of IgG levels. Among the 22 cases of IgG4-RSP reported by Sbeih et al. (10), 13 patients had serum IgG4 of >135 mg/dL. These authors considered that pre-treatment serum IgG4 levels may be used as a marker for the diagnosis of IgG4-RSP. However, the presence of tumors, infections and autoimmune diseases is often accompanied by an increase in serum IgG4. Thus, measurement error would be inevitable. Therefore, the increase in serum IgG4 may only be a moderately effective biomarker for the diagnosis of IgG4-RD or IgG4-RSP (62, 63). From another perspective, serum IgG4 levels may be a viable biomarker to assess the risk of recurrence in IgG4-RD patients, and this relationship was supported by the disease activity score or responder index (64).

The intrathecal synthesis of IgG4 in the CSF may also be used as a reference (8, 13, 16, 26, 33, 39). Della-Torre et al. (13, 16) reported that in the CSF IgG4 index, the IgG4 CSF/IgG4 serum-to-albumin CSF/albumin serum ratio is a reliable marker for intrathecal IgG4 synthesis (normal range: 0.25-0.91). They demonstrated that IgG4Loc of >0.47 is capable of differentiating between IgG4-RHP and inflammatory pachymeningitis, with 100% sensitivity and 100% specificity. Furthermore, the CSF IgG4 level may be a better indicator of disease activity, but regular spinal puncture would be necessary, and the test must be performed before the immunosuppressive therapy (34).

### Radiological diagnosis

**Figure 7:**
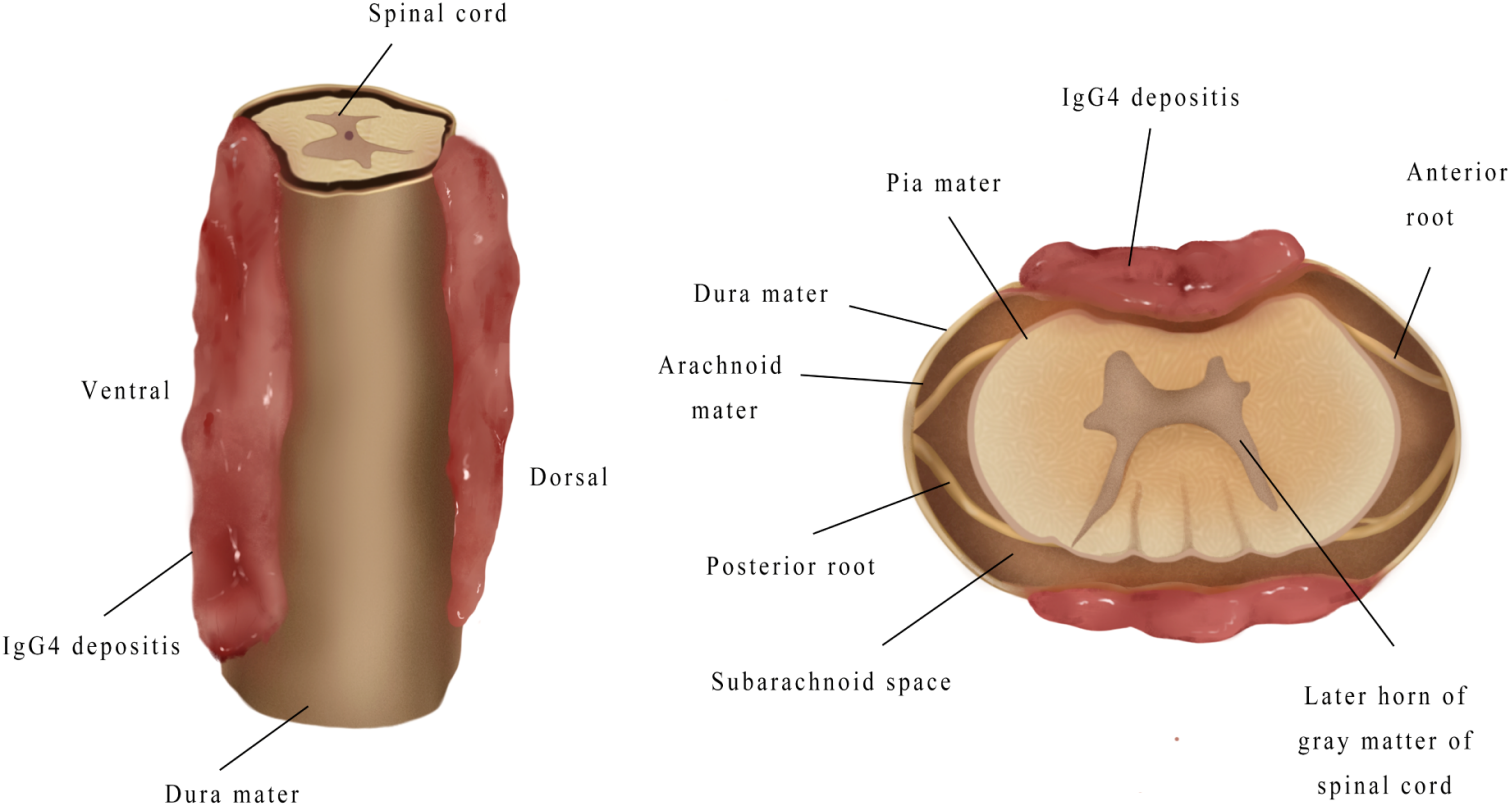
When mass grew on the ventral side, this tended to be thicker, and compressed the spinal cord more severely, when compared to the mass on the dorsal side.

MRI is the most common imaging modality for the diagnosis of IgG-RHP, because this allows for the clear visualization of lesions in the optic chiasm, nerve roots and skull base (55). The present literature review revealed that the characteristic MRI manifestations of IgG4-RSP may include the following: (1) ribbon-shaped masses that commonly involve the cervical and thoracic dura, (2) homogeneous and dorsal lesions on MRI (**Figure 6**), and (3) hypointense signals on T1WI and T2WI, with homogeneous enhancement on T1W1 with gadolinium. In addition, compared to the other two types, homogeneous patients are more likely to have progressive neurological symptoms (**Figure 7**).

However, the specific MRI manifestations of IgG-RHP require further definition. The optimal approach would be to combine the clinical symptoms, radiological manifestations, serological and CSF tests, and pathological outcomes for a definite diagnosis of IgG-RHP. Computed tomography (CT) can also accurately assess the bone involvement in patients with IgG4-RSP. A recent research demonstrated that FDG PET-CT is important in the assessment of IgG-RD, because this can detect unknown sites that are involved, and may be used to monitor the disease activity and therapeutic response of metabolic diseases (55).

Based on the above discussion, it can be considered that IgG4-RSP should be highly suspected when the following conditions are present: (1) progression of symptoms of neurological injury; (2) the characteristic MRI manifestations of IgG4-RSP (refer to the Discussion section above); (3) serum IgG4 of >135 mg/d; (4) CSF abnormalities in the IgG4 index (normal range: 0.25-0.91) with IgG4Loc of >0.47.

### Treatment

Corticosteroids are recognized agents used as the first-line treatment for IgG4-RD (65), and these drugs have up to 98% effectiveness in the early stages of the disease (41). The commonly used corticosteroid treatments are prednisolone and methylprednisolone (66). Although there is no consensus on the optimal treatment dose and duration, most patients respond well to therapy within two weeks of treatment. Li et al. (67) previously used corticosteroids at 20-60 mg/d for 2-4 weeks for the treatment of IgG4-RHP, and the dose was reduced in the subsequent months and years. Corticosteroid treatment also played a role in suppressing recurrence in previous case reports, which is consistent with report in the study conducted by Levraut et al. (34). However, the drug resistance and multiple complications associated with long-term use may limit its use (67).

Steroid-sparing agents, such as rituximab, methotrexate, cyclophosphamide and azathioprine, are advantageous, when compared to corticosteroid treatment, in terms of safety, and these may be used as a second-line treatment for IgG-RD (31). Rituximab is preferred for the treatment of IgG4-RD and IgG4-RHP (34, 64, 68). Hart et al. (69) reported the use of rituximab treatment for IgG4-related autoimmune pancreatitis. The regimen consisted of four weeks of rituximab (i.v.) at 375 mg/m2, weekly, followed by two years of injection every 2-3 months. This treatment resulted in an 83% relief rate. Among the IgG4-RSP cases reported, it was noted that patients who received steroid-sparing agents generally had better outcomes on initial treatment, or inhibition of relapse. Similar to corticosteroid treatment, the optimal treatment dose and duration have not been clarified.

The present study revealed that patients with recurrence responded well to certain doses of corticosteroid treatment and steroid-sparing agents. Focus was given on the use of steroid-sparing agents (11, 20, 22, 33, 40). It was noted that one patient had suspected recurrence (20). This patient had a subcutaneous mass on the left side of the neck at eight months after surgery. After three months of prednisone treatment, the mass was resolved. This result indicates that the recurrence of IgG4-RSP may not occur at the primary site. Therefore, whole-body systemic examination is particularly important for the initial disease, and any recurrences.

Timely surgical decompression and mass resection are required when a patient has severe neurological dysfunction, or a progressive neurological disorder. As stated earlier in the present study, the cumulative location of the mass is correlated with this symptom. However, despite the prompt surgical treatment given, there was still a poor prognosis in the short term, as noted in the case reported by Kim et al. (17). The reason was that the poor prognosis may be associated to an injury in the blood-supplying branches of the radiculomedullary arteries, such as the Adamkiewicz artery. The high tension and traction of the spinal cord during surgical resection may also be a factor. In addition, Sharma et al. (43) reported a male patient with homogeneous IgG4-RSP. The intraoperative results in this study revealed that the dorsal components of homogeneous lesions generally resulted in dural adhesions. Therefore, it is very important to clearly distinguish the mass position from normal anatomical locations. The present study presented a rare intramedullary case of a patient who did not receive surgical intervention. This patient was treated with corticosteroids and azathioprine, and there was effective improvement in the patient’s neurological function.

Overall, it was considered that when IgG4-RSP is accompanied by progressive neurological symptoms caused by definite spinal cord compression, surgical decompression is necessary. However, the present research findings suggest that long-term maintenance therapy with corticosteroid treatment and/or steroid-sparing agents is essential for this population.

## Limitations

First, the present study was a review of the literature on this topic, and was open to considerable subjectivity on the part of the authors. Second, a quality assessment of the included literature was not performed due to the very small number of reported cases of IgG4-RSP worldwide. Third, no systematic statistical analysis was performed due to the small sample size and limited data. However, it is hoped that this literature review deepens the present understanding of IgG4-RSP as a rare disease.

## Conclusion

Progressively deteriorating neurological symptoms can be used as a reference for the diagnosis of IgG4-RSP. The MRI manifestations for lesion morphology, and the site and signal intensity may also be employed as predictive markers. Timely surgical decompression combined with immunosuppressive medications may result in satisfactory therapeutic outcomes. During long-term follow-ups, maintenance therapy with a certain dose of corticosteroids and steroid-sparing agents can effectively reduce the risk of recurrence. Since IgG-RD may involve multiple organs, a whole-body systemic examination is necessary after a definite diagnosis of spinal cord compression.

## Data availability statement

The original contributions presented in the study are included in the article/supplementary material. Further inquiries can be directed to the corresponding author.

## Ethics statement

The studies involving human participants were reviewed and approved by the Medical Ethics Committee of the China-Japan Union Hospital of Jilin University. The patients/participants provided their written informed consent to participate in this study. Written informed consent was obtained from the individual(s) for the publication of any potentially identifiable images or data included in this article.

## Author contributions

FY: Writing and preparation of the original draft, and data curation; ZL: Writing and preparation of the original draft, and data curation; YBZ: Created the figures and tables; PL: data curation; YHZ: Created the figures and tables; QZ: Provided valuable comments; BZ: Writing, review and editing of the manuscript. All authors contributed to the article and approved the submitted version.
